# Time to CT scan for patients with acute severe neurological symptoms: a quality assurance study

**DOI:** 10.1038/s41598-022-19512-x

**Published:** 2022-09-10

**Authors:** Pernille Pape, Alice Herrlin Jensen, Ove Bergdal, Tina Nørgaard Munch, Søren Steemann Rudolph, Lars Simon Rasmussen

**Affiliations:** 1 Department of Anaesthesia, Centre of Head and Orthopaedics, Rigshospitalet, University of Copenhagen, Inge Lehmanns Vej 6, Section 6011, DK-2100 Copenhagen, Denmark; 2 Department of Neurosurgery, Neuroscience Centre, Rigshospitalet, University of Copenhagen, Copenhagen, Denmark; 3 Department of Epidemiology Research, Statens Serum Institut, Copenhagen, Denmark; 4 Department of Clinical Medicine, University of Copenhagen, Copenhagen, Denmark

**Keywords:** Diagnosis, Computed tomography

## Abstract

Emergent brain computed tomography (CT) scan allows for identification of patients presenting with acute severe neurological symptoms in whom medical and surgical interventions may be lifesaving. The aim of this study was to evaluate if time to CT from arrival at the emergency department exceeded 30 min in patients admitted with acute severe neurological symptoms. This was a retrospective register-based quality assurance study. We identified patients admitted to the emergency department with acute severe neurological symptoms between April 1st, 2016 and September 30th, 2020. Data were retrieved from the registry of acute medical team activations. We considered that time to CT from arrival at the emergency department should not exceed 30 min in more than 10% of patients. A total of 559 patients were included. Median time from arrival at the emergency department until CT scan was 24 min (IQR 16–35) in children (< 18 years), 10 min (IQR 7–17) for adults (18–59 years), and 11 min (IQR 7–16) for elders (> 60 years). This time interval exceeded 30 min for 8.2% (95% CI 6.1–10.9) of all included patients, 35.3% of children, 5.9% of adults, and 8.6% of elders. No children died within 30 days. The 30-day mortality was 21.3% (95% CI 16.4–27) in adults, and 43.9% (95% CI 38.2–49.8) in elders. Time from arrival at our emergency department until brain CT scan exceeded 30 min in 8.2% of all included patients but exceeded the defined quality aim in children and could be improved.

## Introduction

Timely interventions may be lifesaving in patients with acute severe neurological symptoms, and the initial work-up of such patients is critical to establish a correct diagnosis. The initial focus is to diagnose highly time-critical conditions such as ischaemic stroke, subarachnoid haemorrhage (SAH), intracerebral haemorrhage (ICH), and metabolic derangements^1^. Without neuroimaging these conditions cannot be reliably distinguished. Rapid and accurate diagnosis using computed tomography (CT) scan of the brain allows for identification of patients that may benefit from neurosurgical or medical interventions.

Even though several guidelines recommend emergent CT scan of the brain as soon as possible after admission only few studies evaluate the prognostic importance of rapid imaging^2^. An emergent CT scan is indicated if the patient presents with loss of consciousness (Glasgow Coma Scale score (GCS) < 13), sudden onset of focal neurological abnormalities, or acute onset of severe headache. The updated American Heart Association/American Stroke Association 2018 guidelines set a door-to-imaging time of 25 min as a best practice strategy for the initial management of patients with suspected stroke^3^. Danish guidelines for patients suspected of SAH recommend a CT scan of the brain within one hour of symptom onset^4–6^.

The primary aim of this quality assurance study was to determine the proportion of patients with acute severe neurological symptoms at admission undergoing a CT scan of the brain within 30 min of arrival to the emergency department (ED). Further, we considered that 30 min from hospital admission to CT scan should not be exceeded in more than 10% of these patients. Secondarily we determined the proportion of patients with a neurosurgeon present and time to neurosurgical intervention.

## Methods

### Design and setting

This was a retrospective register-based quality assurance study conducted at Rigshospitalet in Copenhagen, which is a tertiary university hospital with a level-1 Trauma Centre and Emergency Department.

The study was approved by the hospital management of Rigshospitalet. According to Danish law, no ethics committee approval or informed consent is required for quality assurance studies. The study was conducted in accordance with the Declaration of Helsinki.

### Patient selection

Activation of the acute medical team in our ED is based on prehospital findings including resuscitated cardiac arrest, suspected ruptured aortic aneurism, major bleeding, and a suspicion of time critical neurological or neurosurgical conditions not caused by trauma. These time critical neurological or neurosurgical conditions are classified as “acute severe neurological symptoms” in the ED database. We included patients admitted to the ED at Rigshospitalet presenting with acute severe neurological symptoms between April 1^st^, 2016 and September 30^th^, 2020. We excluded patients without a valid social security number and patients transferred after primary evaluation at another hospital.

### Data collection

Data were retrieved from the ED database containing data from patient charts from all patients admitted to the ED. Data are prospectively collected by the attending clinician using a data collecting sheet and are then stored in the approved research web application, Research Electronic Data Capture (REDCap).

The data extracted included age, gender, comorbidities (neurological comorbidity, obesity, hypertension), and medication (antihypertensives, anticoagulants). We defined neurological comorbidity as previously diagnosed SAH, ICH, ischaemic stroke, epilepsy, and sequelae from head trauma. Data on prehospital information included symptoms and findings (paresis, convulsions, vomiting, unequal pupil size, GCS, cardiac arrest, and vital signs) and interventions (hypertonic saline, haemostatic agents, and airway management). Data on admission to our ED included time of arrival, time of CT scan, medical specialties present in the ED, and time of neurosurgical intervention (defined as time of incision). In addition, we collected information about International Classification of Diseases (ICD-10) discharge diagnoses, mortality during admission, and 30-day mortality. For deceased patients, electronic patient charts were screened for consideration of organ donation and for completed organ harvest. ICD-10 discharge diagnoses were sub-grouped as SAH, ICH, ischaemic stroke, brain tumour, convulsions, headache, subdural haematoma (SDH), intoxication, infection, and “other” (Appendix 1).

If discrepancy was found between information from the registry and information from the electronic patient file, data were corrected according to the electronic patient file.

### Statistics

Continuous variables were reported as medians with interquartile ranges (IQR). Categorical data were reported as numbers (percent) with a 95% confidence interval (CI) where relevant. We set a quality aim that time from hospital admission to brain CT scan should not exceed 30 min in more than 10% of patients. With four or fewer patients out of 100 exceeding 30 min before undergoing CT scan we would be 95% sure to have reached our quality aim.

In the analyses we stratified patients according to age: children (< 18 years), adults (18–59 years), and elders (≥ 60 years). We also compared patients according to CT scan of the brain within or after 30 min, and according to prehospital findings. Differences and odds-ratios were reported with 95% CI. All statistical analyses were performed using SAS studio 3.8 (Enterprise Edition).

### Ethics approval and consent to participate

No informed consent or ethics approval is needed for quality assurance projects.

## Results

**Figure 1:**
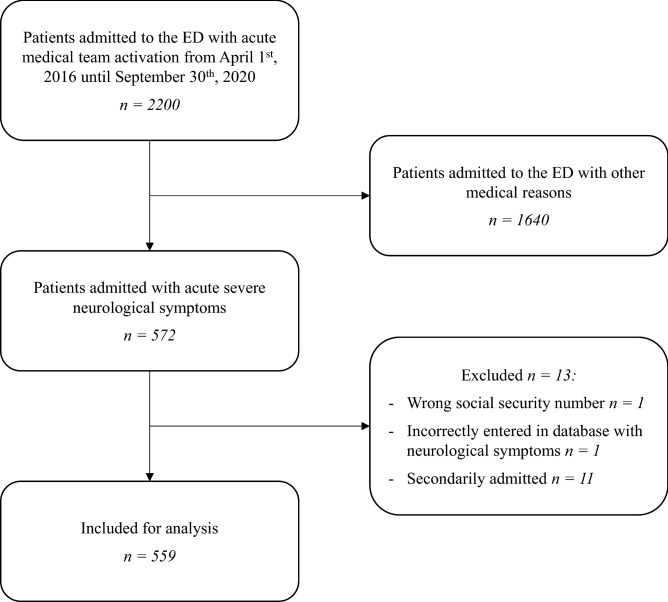
Flowchart of patients admitted to the ED with activation of the acute medical team.

A total of 2200 patients were admitted to the ED with acute medical team activation during the period April 1st, 2016, until September 30th, 2020. During this period 572 patients were admitted with acute severe neurological symptoms. We excluded 13 patients (no valid social security number, incorrect registration in the database with acute severe neurological symptoms, and secondary transfer from another hospital), resulting in a cohort of 559 patients (Fig. 1).

Patient median age was 61 years (IQR 49–73) of which 54.4% were male and 24.2% had neurological comorbidity. Characteristics of patients according to age are presented in Table 1. The most frequent prehospital findings were paresis in children (61.5%) and loss of consciousness (GCS ≤ 8) in adults (48.4%) and elders (68.5%) (Table 2).

**Table 1:** Characteristics of patients admitted at the ED with acute severe neurological symptoms. *n* = 559*.

	Children (< 18) *n* = 19	Adults (18–59) *n* = 244	Elders (≥ 60) *n* = 296
Age (years)	7 (2–12)	49 (40–54)	72 (66–79)
Gender (male)	12 (63.2%)	141 (57.8%)	151 (51%)
ASA score (I/II/III/IV)	15/1/3/0	52/117/67/8	16/95/177/8
**Comorbidities**			
Neurological comorbidity *(n* = *557)*	2 (11.1%)	54 (22.2%)	79 (26.7%)
Obesity (BMI > 30 kg/m^2^) *(n* = *552)*	0	40 (16.6%)	39 (13.4%)
Hypertension *(n* = *554)*	0	61 (25.2%)	172 (58.7%)
**Medication**			
Antihypertensives *(n* = *555)*	0	51 (21%)	164 (56%)
Anticoagulants *(n* = *551)*	0	19 (7.9%)	126 (3.3%)

*Number of patients shown when characteristic is not recorded for all patients.

Data represented as frequencies (percent) or medians (interquartile range).

*ASA score* American Society of Anesthesiologists Physical Status Classification System, *BMI* Body Mass Index.

**Table 2:** Prehospital symptoms, findings, and interventions in patients with acute severe neurological symptoms. *n* = 559*.

	Children (< 18) *n* = 19	Adults (18–59) *n* = 244	Elders (≥ 60) *n* = 296
**Prehospital findings**			
Glasgow coma scale score (3–15)	12 (5–14)	9 (3–14)	6 (3–10)
Glasgow coma scale score ≤ 8	4 (44.4%)	107 (48.4%)	185 (68.5%)
Paresis (*n* = 498)	8 (61.5%)	46 (21%)	72 (27.1%)
Convulsions (*n* = 514)	5 (35.7%)	65 (28.9%)	79 (28.7%)
Vomiting (*n* = 545)	9 (50%)	80 (33.6%)	79 (27.3%)
Unequal pupil size (*n* = 506)	1 (6.7%)	53 (24%)	97 (35.9%)
Cardiac arrest	0	8 (3.3%)	15 (5.1%)
**Blood pressure (mmHg)**			
Systolic (*n* = 493)	120 (111–127)	156 (132–195)	173 (140–209)
Diastolic (*n* = 466)	78 (65–90)	94 (80–110)	95 (78–110)
Heart Rate (BPM) (*n* = 472)	90 (77–107)	80 (65.5–100)	82 (70–100)
Oxygen saturation (%) (*n* = 464)	99 (96–100)	98 (96–100)	96 (92–99)
Respiratory rate pr. min. (*n* = 401)	18 (18–26)	16 (12–20)	16 (12–20)
**Prehospital interventions**			
Hypertonic saline	1 (5.3%)	26 (10.7%)	58 (19.6%)
Haemostatic agents	0	7 (2.9%)	13 (4.4%)
Tracheal intubation	1 (5.3%)	89 (36.5%)	153 (51.7%)
Oxygen by mask	2 (10.5%)	39 (16%)	64 (21.6%)
Oxygen by nasal cannula	0	22 (9%)	21 (7.1%)

*Number of patients shown when characteristic was not recorded for all patients.

Data represented as frequencies (percent) or medians (interquartile range).

CT scan was performed in 17 children (89.5%), 239 adults (98.0%) and 291 elders (98.3%) with a median time from arrival at the ED until CT scan of 24 min (IQR 16–35), 10 min (IQR 7–17) and 11 min (IQR 7–16), respectively (Table 3). The proportion of patients with time to CT above 30 min was 8.2% (95% CI 6.1–10.9) overall, 35.3% (95% CI 14.2–61.8) of children, 5.9% (95% CI 3.2–9.6) of adults and 8.6% (95% CI 5.6–12.4) of elders.

**Table 3:** ED interventions, diagnoses, and outcome in patients with acute severe neurological symptoms. *n* = 559.

	Children (< 18) *n* = 19	Adults (18–59) *n* = 244	Elders (≥ 60) *n* = 296
CT scan performed	17 (89.5%)	239 (98.0%)	291 (98.3%)
Time from arrival to CT scan (min.)	24 (16–35)	10 (7–17)	11 (7–16)
Time from arrival to CT scan > 30 min	6 (35.3%) (14.2–61.8)	14 (5.9%) (3.2–9.6)	25 (8.6%) (5.6–12.4)
Neurosurgeon present in ED	2 (10.5%)	64 (26.2%)	88 (29.7%)
Underwent neurosurgical intervention	3 (15.8%)	56 (23%)	61 (20.6%)
Time to neurosurgical intervention (min.)	90 (59–109)	84 (66.5–103)	73 (60–89)
**ICD-10 diagnosis codes**			
Intracranial haemorrhage	3 (15.8%)	56 (23%)	95 (32.1%)
Subarachnoid haemorrhage	1 (5.3%)	47 (19.3%)	46 (15.5%)
Convulsions	5 (26.3%)	20 (8.2%)	34 (11.5%)
Headache	2 (10.5%)	43 (17.6%)	10 (3.4%)
Subdural haematoma	0	6 (2.5%)	25 (8.5%)
Ischaemic stroke	2 (10.5%)	10 (4.1%)	18 (6.1%)
Intoxication	0	8 (3.3%)	6 (2%)
Infection	0	4 (1.6%)	8 (2.7%)
Brain tumour	0	6 (2.5%)	2 (0.7%)
Other diagnosis	2 (10.5)	34 (13.9%)	44 (14.9%)
Undetermined	4 (21.2%)	10 (4.1%)	8 (2.7%)
**Outcome**			
Died in ED	0	2 (0.8%)	7 (2.4%)
Died during subsequent admission	0	51 (20.9%)	128 (43.2%)
30-day mortality	0	52 (21.3%)	130 (43.9%)
Consideration of organ donation	–	34 (64.2%)	59 (43.7%)
Organ harvest performed	–	11 (20.8%)	7 (5.2%)

Data represented as medians (interquartile range) or frequencies (percent) and 95% confidence interval where relevant.

A neurosurgeon was present in the ED for 10.5% of children, 26.2% of adults and 29.7% of elders. ICH was the most frequent discharge diagnosis in all age groups. No children died within 30 days whereas the 30-day mortality was 21.3% (95% CI 16.4–27) for adults and 43.9% (95% CI 38.2–49.8) for elders. Organ donation was mentioned in 64.2% of deceased adults and 43.7% of deceased elders (Table 3).

We found no difference in the proportion with a neurosurgeon present or in time until neurosurgical intervention according to time to brain CT below vs. above 30 min. Likewise, there was no significant difference in 30-day mortality (Table 4).

**Table 4:** Contact to neurosurgeon and outcome according to time to CT below or above 30 min. *n* = 547.

	Time from arrival to CT scan < 30 min *n* = *502*	Time from arrival to CT scan > 30 min *n* = *45*	Odds Ratio
Neurosurgeon present in ED	140 (27.9%)	12 (26.7%)	
Time to neurosurgical intervention (min.)	77 (64–97)	89 (64–105)	
30-day mortality	163 (32.5%) (28.4–36.8)	14 (31.1%) (18.2–46.7)	0.94 (0.49–1.82)

Data represented as medians (interquartile range) or frequencies (percent) with 95% Confidence interval (CI) for mortality. Difference reported as odds ratio with 95% CI for mortality.

We also found no significant difference in the proportion of patients with time to CT above 30 min according to a prehospital GCS ≤ 8, or presence of either convulsions or unequal pupil size (Table 5). A neurosurgeon was present in the ED in a higher proportion of patients with prehospital GCS ≤ 8 (35.1% vs. 18.1%) and in patients with prehospital unequal pupil size (43.1% vs. 21.4%).

**Table 5:** Interventions in patients admitted with acute severe neurological symptoms according to prehospital findings.

	Prehospital GCS > 8 *n* = 204	Prehospital GCS ≤ 8 *n* = 296	Difference
Time from arrival to CT scan (min.)	10 (7–15)	11 (7–17)	
Time from arrival to CT scan > 30 min	10 (5%) (2–8.1)	26 (8.9%) (5.7–12.2)	3.9% (-0.6–8.4)
Neurosurgeon present in ED	37 (18.1%) (12.9–23.4)	104 (35.1%) (29.7–40.6)	17.0% (9.4–24.6)
Underwent neurosurgical intervention	30 (14.7%)	82 (27.7%)	
Time to neurosurgical intervention (min.)	100 (67–144)	73 (64–90)	
30-day mortality	21 (10.3%) (6.5–15.3)	153 (51.7%) (45.8–57.5)	41.4% (34.3–48.5)

	No convulsions *n* = 365	Prehospital convulsions *n* = 149	Difference
Time from arrival to CT scan (min.)	10 (7–16)	10.5 (7–19)	
Time from arrival to CT scan > 30 min	23 (6.4%) (4.1–9.5)	15 (10.3%) (5.9–16.4)	3.8% (-1.7–9.4)
Neurosurgeon present in ED	111 (30.4%) (25.7–35.1)	33 (22.2%) (15.5–28.87)	8.3% (0.1–16.4)
Underwent neurosurgical intervention	76 (20.8%)	36 (24.2%)	
Time to neurosurgical intervention (min.)	77 (67–99)	78 (61–94)	
30-day mortality	120 (32.8%) (28.1–38)	52 (34.9%) (27.3–43.1)	2.0% (-7.0–11.1)

	Prehospital equal pupil size *n* = 355	Prehospital unequal pupil size *n* = 151	Difference
Time from arrival to CT scan (min.)	10 (7–17)	10 (6–15)	
Time from arrival to CT scan > 30 min	28 (8.1%) (5.4–11.4)	9 (6.1%) (2.8–11.3)	1.9% (− 2.9–6.7)
Neurosurgeon present in ED	76 (21.4%) (17.1–25.7)	65 (43.1%) (35.2–50.9)	21.6% (12.7–30.6)
Underwent neurosurgical intervention	62 (17.5%)	48 (31.8%)	
Time to neurosurgical intervention (min.)	85 (64–109)	73 (65–83)	
30-day mortality	90 (25.4%) (20.9–30.2)	82 (54.3%) (46–62.4)	29.0% (19.8–38.1)
Data represented as medians (interquartile range) or frequencies (percent) with 95% confidence interval where relevant. Differences reported with 95% confidence interval			

The proportion of patients with time to CT above 30 min according to discharge diagnosis is shown in Table 6.

**Table 6:** Interventions and outcome according to international classification of diseases-10 diagnoses.

	Intracranial haemorrhage *n* = 154	Subarachnoid haemorrhage *n* = 94	Convulsions *n* = 58	Headache *n* = 55	Subdural haematoma *n* = 30	Ischaemic stroke *n* = 30	Intoxication *n* = 14	Infection *n* = 11	Brain tumour *n* = 7
Time from arrival to CT scan (min.)	10 (7–15)	9 (7–12)	14.5(8–22)	8 (5–16)	10.5 (6–15)	14 (10–27)	19 (10–22)	12 (11–29)	7 (4–12)
Time from arrival to CT scan > 30 min	10 (6.5%)	1 (1.1%)	7 (12.1%)	2 (3.6%)	2 (6.7%)	6 (20%)	1 (7.1%)	2 (18.2%)	0
Neurosurgeon present in ED	80 (52%)	47 (50%)	0	1 (1.8%)	18 (58%)	1 (3.3%)	1 (7.1%)	1 (8.3%)	1 (12.5%)
Underwent neurosurgical intervention	55 (35.7%)	51 (54.3%)	0	0	9 (29%)	3 (10%)	0	0	1 (12.5%)
Time to neurosurgical intervention (min.)	80 (65–100)	73 (62.5–98.5)	0	0	76 (69–83)	105 (73–188)	0	0	47
30-day mortality	96 (62.3%)	33 (35.1%)	2 (3.4%)	0	22 (71%)	9 (30%)	1 (7.1%)	2	
(16.7%)	0								

Data represented as medians (interquartile range) or frequencies (percent).

## Discussion

The main finding in this study was that time from arrival at the ED until CT scan of the brain exceeded 30 min in 8.2% (95% CI 6.1–10.9) overall, 35.3% (95% CI 14.2–61.8) of children, 5.9% (95% CI 3.2–9.6) of adults and 8.6% (95% CI 5.6–12.4) of elders with acute severe neurological symptoms. We found no association between time to CT scan above 30 min or time to neurosurgical intervention and 30-day mortality among patients with acute severe neurological symptoms.

One important strength of this study is that there were no missing data regarding the primary outcome of time to CT. Likewise, we had complete information about outcome and final diagnoses. Some prehospital variables and baseline characteristics had missing data, and the analyses based on these should be interpreted with caution.

One limitation of this retrospective study is that data were not collected specifically for this study and the time of CT scan may not have been documented exactly in all cases. When possible, we verified time of CT scan in the electronic patient imaging system, but for patients admitted prior to November 2016, this was not always possible as the new electronic patient file system was implemented at that time. This introduces a risk of inaccuracy.

The database did not contain all variables that may be of importance. We had no information about timing of neurosurgical consultation as it was only recorded whether a neurosurgeon was present in the ED during the workup. This invites a cautious interpretation of difference in the proportion of neurosurgical consults according to time to CT.

We estimated that 100 patients would have been sufficient to exclude that 10% or more exceeded the defined level of quality in case no more than 4 patients exceeded 30 min to CT scan. Our database included data since 2016 and with 559 eligible patients. Still, the upper limit of the 95% CI was 10.9% for the proportion with time to CT above 30 min.

The categorisation of patients into the three age groups was somewhat arbitrary. Another result could have been found if children had been defined as those younger than 15 years, for instance, but in our system all decision-making is different for minors younger than 18 years and we already had fewer patients in this group. The analyses would therefore be even less reliable if that age limit was lowered. Elders were defined as age above 60 years similar to what has been used in other studies^7,8^.

Patients are referred to the ED at our hospital prehospitally by an anaesthesiologist attending the physician-staffed mobile unit or by the physician at the emergency medical coordination centre. Triage follows regional guidelines, ensuring that the most critically ill patients who may benefit from highly specialised treatment, are brought directly to our hospital. Elderly patients are at risk of undertriage and less likely to trigger a trauma team activation^9,10^. Elderly patients are more often referred to a local emergency department and secondarily admitted to our hospital after initial CT scan of the brain, if specialised treatment is indicated. Undertriage of elderly patients may cause a selection bias, as we excluded secondarily admitted patients. This could result in an underestimation of time to CT scan because time to CT could be longer in elderly with diffuse symptoms. Subdural haematoma was found in a number of patients with no suspicion of trauma, but it is not always possible to establish what the mechanism of injury is when assessed in the prehospital setting.

In a study evaluating delays of stroke thrombolysis pathways, mean time to CT was 60 min^11^. We found a median time to CT of 24 min for children, 10 min for adults and 11 min for elders. A significant decrease in in-hospital mortality was found after a focused effort resulting in a reduction of time from arrival at hospital until thrombolysis in patients with ischaemic stroke^12^. We found no significant difference in mortality according to time to CT scan, but ICH constituted a major group and fewer had ischaemic stroke.

A prospective cohort study showed that paediatric stroke differs from adults in clinical presentation and stroke is rare in children. Delays of CT scan in children may result from the initial focus on multiple other and more common diagnoses. Furthermore, children may need anaesthesia to facilitate high quality CT images by avoiding movement during scan^13,14^. Finally, clinicians may be hesitant to perform CT scan in children because of the radiation exposure^15^. This could possibly delay relevant interventions and even lead to a poorer outcome.

We considered 30 min from arrival until CT scan to be clinically relevant at our ED. The mortality analysis according to time to CT does not allow firm conclusions as illustrated by our wide confidence intervals. Even though we did not observe a difference in mortality, we still suggest attention should be given to keep low door-to-imaging times to facilitate fast diagnosis and fast treatment.

We found that 64.2% of deceased adults and 43.7% of deceased elders had documentation in their patient file of consideration of organ donation. In a previous retrospective cohort study of all deaths in intensive care units and emergency departments in four Canadian hospitals with 2931 deceased patients found that documentation of organ donation discussion rose from 7.9% in 2008 to 26.3% in 2010. Most patients died of cardiac arrest (39.2%) followed by ICH (16.3%) in their study^16^.

Globally there is a continuous organ shortage^17^. Documenting consideration of organ donation in critically ill or dying patients should get continuous attention as we may be missing potential organ donors.

## Conclusions

In conclusion, time from arrival at our ED until brain CT scan exceeded 30 min in 8.2% (95% CI 6.1–10.9) of patients admitted to the ED with acute severe neurological symptoms. Time from arrival until CT scan did not exceed our quality aim in adults or elders but could be improved for children.

## Supplementary Information

Supplementary Information.

## Data Availability

The datasets used and analysed during the current study are available from the corresponding author on reasonable request.

## Abbreviations

CT: Computed tomography
ED: Emergency department
SAH: Subarachnoid haemorrhage
ICH: Intracerebral haemorrhage
GCS: Glasgow coma scale score
REDCap: Research electronic data capture
ICD: International classification of diseases
SDH: Subdural haematoma
IQR: Interquartile range
CI: Confidence interval
OR: Odds ratio
