# Supplementary material for: Time to CT scan for patients with acute severe neurological symptoms: a quality assurance study
Source: Sci Rep. 2022 Sep 10;12:15269. doi: 10.1038/s41598-022-19512-x (PMC9464221; doi:10.1038/s41598-022-19512-x)
Supplement: Supplementary file 1 — Supplementary Information. [file 41598_2022_19512_MOESM1_ESM.docx]

# Appendix

| **Appendix 1:** Contents of the subgroup “other diagnosis” out of all diagnoses in patients admitted with acute severe neurological symptoms. *n = 559* | |
| --- | --- |
| Diagnosis code | Frequency |
| Unresolved | 22 (3.9%) |
| Syncope | 8 (1.4%) |
| Cardiac arrest | 8 (1.4%) |
| ST-Segment elevation myocardial infarction | 6 (1.1%) |
| Aortic dissection | 5 (0.9%) |
| Abstinence convulsions | 3 (0.5%) |
| Acute kidney insufficiency | 2 (0.4%) |
| Aortic aneurysm | 2 (0.4%) |
| Carotid stenosis | 2 (0.4%) |
| Concussion | 2 (0.4%) |
| Hypoglycaemia | 2 (0.4%) |
| Hyponatraemia | 2 (0.4%) |
| Internal carotid artery dissection | 2 (0.4%) |
| Chronic Obstructive Pulmonary Disease | 2 (0.4%) |
| Reversible cerebral vasoconstriction syndrome | 2 (0.4%) |
| Atrioventricular block | 1 (0.2%) |
| Aneurism | 1 (0.2%) |
| Aortic stenosis | 1 (0.2%) |
| Aplastic anaemia | 1 (0.2%) |
| Anterior spinal artery syndrome | 1 (0.2%) |
| Colon fistula | 1 (0.2%) |
| Deep vein thrombosis | 1 (0.2%) |
| Delirium | 1 (0.2%) |
| Encephalopathy | 1 (0.2%) |
| Skull base fracture | 1 (0.2%) |
| Hypothermia | 1 (0.2%) |
| Ketoacidosis | 1 (0.2%) |
| Pulmonary embolism | 1 (0.2%) |
| Malignant neuroleptic syndrome | 1 (0.2%) |
| Vestibular neuritis | 1 (0.2%) |
| Obesity hypoventilation syndrome | 1 (0.2%) |
| Psychogenic Non-Epileptic Seizures | 1 (0.2%) |
| Posterior reversible encephalopathy syndrome | 1 (0.2%) |
| Post viral encephalitis | 1 (0.2%) |
| Sinoatrial block | 1 (0.2%) |
| Shunt dysfunction | 1 (0.2%) |
| Transient cerebral ischaemia | 1 (0.2%) |
| Vertebral artery dissection | 1 (0.2%) |
| Vestibular neuritis | 1 (0.2%) |
| Iron deficiency anaemia | 1 (0.2%) |
| Overhydration | 1 (0.2%) |
| Data represented as frequencies (percent). | |
